# Appraisal of kinetin spraying strategy to alleviate the harmful effects of UVC stress on tomato plants

**DOI:** 10.1007/s11356-022-19378-6

**Published:** 2022-03-08

**Authors:** Mona F. A. Dawood, Abdelghafar M. Abu-Elsaoud, Mahmoud R. Sofy, Heba I. Mohamed, Mona H. Soliman

**Affiliations:** 1 Botany and Microbiology Department, Faculty of Science, Assiut University, Assiut, 71516 Egypt; 2 Botany Department, Faculty of Science, Suez Canal University, Ismailia, Egypt; 3 Botany and Microbiology Department, Faculty of Science, Al-Azhar University, Nasr City, 11884 Cairo Egypt; 4 Biological and Geological Sciences Department, Faculty of Education, Ain Shams University, Roxy, P.C.11757, Heliopolis Cairo, Egypt; 5 Botany and Microbiology Department, Faculty of Science, Cairo University, Giza, 12613 Egypt; 6 Biology Department, Faculty of Science, Taibah University, Al-SharmYanbu El-Bahr, , Yanbu 46429 Kingdom of Saudi Arabia

**Keywords:** Antioxidants, Kinetin, Metabolites, Tomato, UVC

## Abstract

Increasing ultraviolet (UV) radiation is causing oxidative stress that accounts for growth and yield losses in the present era of climate change. Plant hormones are useful tools for minimizing UV-induced oxidative stress in plants, but their putative roles in protecting tomato development under UVC remain unknown. Therefore, we investigated the underlying mechanism of pre-and post-kinetin (Kn) treatments on tomato plants under UVC stress. The best dose of Kn was screened in the preliminary experiments, and this dose was tested in further experiments. UVC significantly decreases growth traits, photosynthetic pigments, protein content, and primary metabolites (proteins, carbohydrates, amino acids) but increases oxidative stress biomarkers (lipid peroxidation, lipoxygenase activity, superoxide anion, hydroxyl radical, and hydrogen peroxide) and proline content. Treatment of pre-and post-kinetin spraying to tomato plants decreases UVC-induced oxidative stress by restoring the primary and secondary metabolites’ (phenolic compounds, flavonoids, and anthocyanins) status and upregulating the antioxidant defense systems (non-enzymatic antioxidants as ascorbate, reduced glutathione, α-tocopherol as well as enzymatic antioxidants as superoxide dismutase, catalase, ascorbate peroxidase, glutathione peroxidase, glutathione-S-transferase, and phenylalanine ammonia-lyase). Thus, the application of Kn in optimum doses and through different modes can be used to alleviate UVC-induced negative impacts in tomato plants.

**Graphical abstract:**

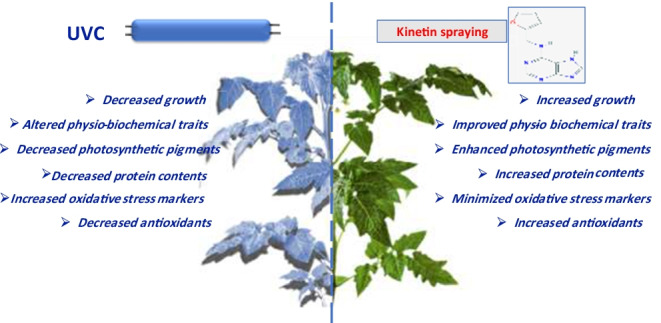

## Introduction

Plants, being sessile creatures, cannot avoid exposure to ecologically induced alterations that seriously impact their growth and performance (Singh et al. 2019). Solar light is essential to plant life; UV radiation is a component of the sunlight that reaches the Earth’s surface affecting the living organisms (Yin and Ulm 2017; Zlatev et al. 2012). Apart from this light, the UV radiation range comprises UVC, UVB, and UVA based on their wavelength range (100–280 nm), 280–315 nm, and UVA 315–400 nm, respectively (Dawood et al. 2021a; Muller-Xing et al. 2014).

UVC is considered the most energetic UV spectrum that is hardly received by living organisms on our planet due to the presence of the stratosphere’s ozone layer (Sgroppo and Sosa 2021). The damaging consequences of UVC radiation nowadays are ascribed to problems of ozone depletion in the stratosphere are gradually exceeding and coined as stressors (Ballare et al. 2011; Biever et al. 2014; Rai and Agrawal 2020). Although the ozone layer in the stratosphere efficiently masks UVC radiation, it minimizes its level reaching Earth’s surface, releasing chlorofluorocarbons into the atmosphere and depleting the ozone layer. Therefore, the increasing trend of the amount of UVC radiation reached to the soil in the future culminates the ecological consequences of increasing UVC on the natural environment and on the agricultural system (Kataria and Guruprasad 2012). The deteriorations of UVC are associated with the production of high-energy free radicals with high absorptivity in tissues causing impairments at molecular levels (ICNIRP 2007). In this regard, excessive UVB and UVC application induced dimerization and ionization of pyrimidines of DNA, causing its damage interfering with protein structure and biosynthesis (Sharma et al. 2017; Tossi et al. 2019), accounting for a serious shift in normal cellular processes in plants (Nawkar et al. 2013).

Exposure to high-energy UVC radiation causes morphological, developmental, and physiological destruction, including leaf area, chlorophyll content, photosynthesis, DNA, membrane lipids, and proteins (Geras’kin et al. 2016; Muller-Xing et al. 2014). UVC radiation in plant cells or organelles induces oxidative stress, and pioneering research has shown that chloroplasts seem to be the most responsive structures (Nawkar et al. 2013; Vanhaelewyn et al. 2020), hence adversely affecting photosynthetic productivity and minimizing crop productivity (Czegeny et al. 2016). Plants have developed sophisticated tolerance levels to mitigate the damage caused by UVC radiation stress, including the implementation of anatomical and morphological features like the thickness of leaves, modification of cuticle composition, and improved synthesis of protective pigments (Germ et al. 2013; Kreslavski et al. 2020; Ullah et al. 2020). The plants’ defense strategy to UVC stress could also implicate the production of phenolic compounds in the extrinsic epidermal tissues, the formation of antioxidant molecules, and the enhanced antioxidant enzymes for attenuating plant susceptibility by UVC radiation (Muller-Xing et al. 2014). Therefore, it is tempting to determine the plant-UVC interaction responses in terms of growth, stress susceptibility traits, and physiological characteristics.

Upgrading the level of plant defense by external protective agents is a common way to ameliorate the negative impacts of UVC radiation stress and other stressors on plants. Plant growth regulators (PGRs) are signaling agents that are widely applied for controlling growth and development activities in crop plants and alleviating the effects of abiotic and biotic stress (Ashry et al. 2018; Dawood et al. 2021b; Sofy et al. 2021d). Among them is kinetin (Kn), a synthetic PGR synthesized from a cytokine, adenine, and implicated in managing the cell expansion and differentiation, leaf, and chloroplast senescence (Agha et al. 2021; Dong et al. 2009). The application of Kn has been reported to decrease the toxic effects by interacting with other PGRs, including salicylic acid, gibberellic acid, jasmonic acid, and abscisic acid (Acidri et al. 2020; Sofy et al. 2020). Although the function of cytokinins in modulating multi-biological pathways during normal cellular metabolism and prohibiting oxidation during stress conditions, their endogenous content was declined during oxidative stress (Zwack and Rashotte 2015). Thus, external spraying of such PGR might be restoring normal cellular metabolism during abiotic stresses or at least attenuating stress-related responses. Several studies applied kinetin extensively as an oxidative stress mitigator and growth promoter (Ahanger et al. 2018; Mohamed et al. 2016; Singh et al. 2019). Furthermore, the application of Kn alleviated the UV-B-induced effects by accumulating phenolics and antioxidants in *Solanum lycopersicum* (Abu-Elsaoud et al. 2008; Amal et al. 2015; Singh et al. 2019). As UVB and UVC are highly energetic radiations, their damaging consequence may be showed similar responses. Therefore, the rationale behind using kinetin in the present study as an alleviator of UVC stress could be tested.

*Solanum lycopersicum* L. (tomato) is among the largest commercially consumed vegetable crops across the globe with high nutrition value (Sofy et al. 2021a). Tomato has high β-carotene, lycopene, flavonoids, and ascorbic acid content and is considered one of the effective anti-oxidative and anti-cancerous fruits to humanity’s service (Naeem et al. 2020). Furthermore, tomato contains essential amino acids, carbohydrates, minerals, vitamins (A, C, and E), and various antioxidants that strengthen the plant defense system (De la Torre-González et al. 2018). Consequently, the environmental warming circumstance indicates that life on Earth is endlessly subjected to powerful UV rays’ dosages. Therefore, the consequences of UV radiation on crops should be thoroughly investigated to better understand physiological and biochemical aspects, ensure relatively high crop yield, and satisfy the increasing population requirement. In addition, amelioration of negative impacts of UVC should be considered as a tool to improve various co-stressor encountered by plants under UVC. Nonetheless, Kn spraying’s effect on alleviating the damaging impacts of UVC stress in tomato plants has not been reported to date. Therefore, the present research work was thus undertaken. Thus, the current investigation’s aim was coined as (1) selecting the most desirable concentration of Kn to alleviate UVC stress and (2) evaluating the physiological and biochemical responses of tomato plants to the optimal Kn concentration applied pre- or post-UVC exposure.

## Materials and methods

### Plant material and experimental setup

Commercial hybrid-F1 841 seeds of tomato (*Lycopersicon esculentum* L.) plants were purchased from Mecca TRADE Co. as Asia Seed co. Lid product, Korea. The cultivars were selected based on their importance as vigor hybrids. It is the most popular and widely cultivated in Upper Egypt, with little physiological history in the literature under biotic/abiotic stresses. Before sowing, tomato seeds were sterilized with HgCl_2_ (0.1%) for 5 min and then rinsed vigorously with distilled water three times. Next, the seeds were sowed in trays filled with 100% peat moss. Then, the trays were kept in a greenhouse at 25 ºC. The trays were fertilized with a water-soluble fertilizer (NPK, 20:20:20) at a rate of 150 mg/L. The seedlings (35 days) were then transplanted to pots (30 cm diameter) filled with 1 kg of clay soil. The experiment was conducted under natural conditions in the Botanical Farm of Botany and Microbiology Department, Faculty of Science, Assiut University, Egypt (27°12’N latitude and 31°09’E longitude; mean day/night temperature, 25/14 °C respectively; relative humidity between 35 and 70%; and the day/night cycle was 14/10 h). Three seedlings of similar lengths and sizes were remained per pot to be used throughout the experiments. Each experimental pot was given a 250-mL full-strength nutrient solution (Hoagland and Arnon 1950).

### UVC treatment

Pots were divided into three groups; group 1 was not treated with UVC radiation (control). Group 2 was subjected to UVC radiation only-the third group containing five sub-groups (Kn application). Tomato plants (35 days) were subjected to UVC stress using UVC lamps (Philips, 20 W) for 20 min. The plants have been arranged 40 cm away from the UVC lamp placed at the top of the plants.

### Selecting the most appropriate concentration of kinetin to mitigate UVC stress

The third group containing five sub-groups was sprayed with different concentrations of Kn (0.05, 0.1, 1, 2, and 3 mM) a day before the exposure to UVC. Then, the plants were sprayed with 20 ml of the corresponding concentration. The plants were exposed to radiation within three days from 11:12 am. Pots were kept under natural humidity, temperature, and sunlight conditions at the Botanical Farm, Botany and Microbiology Department, Faculty of Science, Assiut University, Assiut, Egypt. The experiment was carried out with three replicates for each treatment and was fully randomized. One week after applying the last UVC dose, the plants were harvested to determine the best Kn concentration. Morphological traits (as lengths and weights) of the harvested plants were chosen for this purpose.

### To evaluate the efficacy of kinetin: pre-and post-application of kinetin to UVC-treated plants

The tomato plants were divided into four groups, as shown in Table 1: (1) Reference control group included a sub-group of non-UVC irradiated plants and sub-groups of plants exposed to two doses of UVC by changing the exposure time for 20 and 40 min. (2) The UVC exposure was done for three days between 11:12 am. (03) Kn spraying pre-UVC exposure group: The plants were sprayed with the best Kn concentration (1 mM, 20 mL/pot). The calculated amount of kinetin was dissolved in 5 mL ethanol and then completed to the definite volume. After one day, the plants were subdivided into two sub-groups; one for non-irradiated plants and the other sub-groups of plants exposed to 20 and 40 min of UVC for three days. (4) Kn spraying post-UVC exposure group: The treatments were applied after one day of the last UVC exposure. The plants were subjected to the best concentration of Kn (1 mM, 20 mL/pot). Three pots per treatment were conducted. Fifteen days from the beginning of the experiment, the plants were harvested for morphological and physiological purposes.

### Measurement of growth parameter

Fifteen days after treatment (DAT), morphological characteristics of all treatments have been documented. Five roots were harvested and taken to the laboratory to measure various growth parameters, including plant height, fresh and dry weights, sprinkles and roots, and 72 h after shade drying at room temperature.

### Photosynthetic pigments, anthocyanins, and chlorophyll stability index

The Chl *a*, *b*, and carotenoids contents were determined using the method of (Lichtenthaler 1987); briefly, a sample of 0.2 g of the leaf was suspended for 24 h in 10 mL of prechilled 95% ethyl alcohol. Absorption was taken at different wavelengths: 664 nm, 633 nm, and 452 nm using 95% ethyl alcohol as a blank. The chlorophyll stability index (CSI) was determined according to Murphy (1962) method and calculated as follows:

CSI = (Chl a content of warmed leaves in water (56 ± 1 °C)/ Chl a content of intact fresh leaves) × 100.

To determine anthocyanin levels, 0.5 g of the leaf sample was soaked in 3 mL of acidified methanol (1% v/v HCl) for 12 h in darkness at 4 °C with occasional shaking. The mixture was then centrifuged for 10 min at 14,000 × g at 4 °C. The absorption of the supernatant was read at 530 nm by a spectrophotometer (Mancinelli 1984). The anthocyanin content was expressed as μmolg^−1^ FW using an extinction coefficient of 33.000 mol^−1^ cm^−1^.

### Estimation of stress-induced biomarkers

#### Lipid peroxidation

Malondialdehyde (MDA) content was analyzed in Heath and Packer (1968) techniques to see how much lipid has been oxidized (lipid peroxidation). Briefly, fresh leaves were macerated in trichloroacetic acid (TCA) and centrifuged at 12,000 × g. Next, the supernatant was reacted with thiobarbituric acid in the boiling water bath for 30 min. After cooling the samples, optical absorbance was recorded at 532 nm.

#### Hydrogen peroxide

Leaf samples were extracted in 5% TCA and centrifuged at 11,500 × g for 15 min. The supernatant was mixed with 10 mM phosphate buffer (pH 7.0) and 1 M KI, and absorbance was recorded at 390 nm (Velikova et al. 2000).

##### Superoxide anion

The foliar content of superoxide anion (O_2_^●─^) was determined by extracting in K-phosphate buffer and using the detailed method of Yang et al. (2011). First, the extract was incubated in hydroxylamine hydrochloride. After incubation for 20 min, sulphanilamide and α-naphthyl were added and the optical density was done spectrophotometrically at 530 nm using NO_2_^−^ as a standard curve.

#### Hydroxyl radical

Hydroxyl radicals (OH^●─^) concentration was estimated following Halliwell et al. (1987). Reaction mixture contained deoxyribose, KH_2_PO_4_ buffer (20 mM, pH 7.4), 100 µM FeCl_3_, 104 µM EDTA, 1 mM H_2_O_2_, and 100 µM ascorbate in final 1 mL. After incubation at 37 °C for 1 h, optical density was recorded at 532 nm.

#### Lipoxygenase

Lipoxygenase (LOX; EC, 1.13.11.12) activity was assayed by adopting Doderer et al. (1992) method. Linoleic acid was used as a substrate, and the change in optical density was monitored at 234 nm. The activity was expressed as units mg^−1^ protein, and an extinction coefficient of 25 mM^−1^ cm^−1^ was used for calculation.

### Determination of total soluble carbohydrates, protein, proline, and free amino acids

Total soluble sugar was estimated according to the modified method of (Irigoyen et al. 1992) using an anthrone reagent, and the absorbance was recorded at 625 nm using glucose as a standard. The soluble protein content was estimated following (Lowry et al. 1951) using Folin phenol reagent, and the absorbance was recorded at 700 nm using bovine serum albumin as standard. Bates et al. (1973) method was used to estimate proline. Briefly, 0.5 g of fresh leaves was extracted in 3% sulphosalicylic acid. After centrifugation at 10.000 × g for 10 min, the supernatant was mixed with ninhydrin reagent, and the absorbance was taken at 520 nm. Finally, the method of Rubinstein and Pryce (1959) was used to estimate free amino acids using stannous chloride reagent and boiled in the water bath for 20 min. After cooling, ethanol 50% was placed in the last solution, where the optical density of violet color was performed at 570 nm.

### Extraction and assay of total flavonoids and total phenolic compounds

Zou et al. (2004) method was followed to determine flavonoids. First, 0.5 mL of methanolic extract was mixed with NaOH for 5 min followed by the addition of 0.5 ml 5% NaNO_2,_ which was left for 6 min. Then, to the last mixture, 3 mL of AlCl_3_ was added to the previous mix, leaving it for another 6 min and then adding 2 mL of NaOH (1 N), and the mixture was completed to 5 mL with distilled water. Finally, the developed color was measured spectrophotometrically at 510 nm using quercetin as a standard curve.

Total phenolic content was estimated using the method described by Kofalvi and Nassuth (1995), with minor modifications. First, a 100-μL volume of extract was added to 0.5 mL Folin–Ciocalteu reagent solution and incubated at room temperature for 1 min. Subsequently, 1.5 mL of sodium carbonate solution (20%) was added and incubated for 90 min in the dark at room temperature. Absorbance had been read at 725 nm. Total phenolic content was determined from a calibration curve of gallic acid and expressed as mg g^−1^ dry weight.

### Assay of antioxidant enzymes

Fresh tomato (1.0 g) leaves were extracted in 100 mM phosphate buffer (pH 7.8) containing PVP and EDTA. The homogenate was centrifuged at 15.000 × g for 10 min. The supernatant was used for assaying enzyme activity. The superoxide dismutase activity (SOD; EC 1.15.1.1) was assayed by Misra and Fridovich (1972). The ability of the enzyme to autooxidize epinephrine was recorded at 480 nm. Chen et al. (2000) determined catalase activity (CAT; EC 1.11.1.6), and the disappearance of H_2_O_2_ was monitored at 240 nm for 3 min. Nakano and Asada (1981) method was used to determine ascorbate peroxidase activity (APX; EC 1.11.1.11), where absorbance was recorded at 290 nm for 3 min. One milliliter of the assay mixture contains phosphate buffer (100 mM; pH 7.0), 0.5 mM ascorbic acid, H_2_O_2_, and enzyme extract (100 µL). For calculation, an extinction coefficient of 2.8 mM^−1^ cm^−1^ was used. Glutathione peroxidase (GPX; EC.1.11.1.9) was followed by Flohé and Günzler (1984) method. A reaction mixture of K-phosphate buffer, reduced glutathione, Na_2_HPO_4_, and 5,5-dithiobis-2-nitrobenzoic acid has been mixed with enzyme extract. The absorbance at 412 nm was recorded after 5 min, and the GPX activity was calculated by applying an extinction coefficient of 6.22 mM^−1^ cm^−1^.

GST (EC 2.5.1.18) was assayed by recording the absorbance at 340 nm for 2 min in a 3-mL assay mixture containing 100 mM phosphate buffer (pH 6.5), GSH (1.5 mM), 1-chloro-2, 4-dinitrobenzene (1 mM), and enzyme extract. For calculation extinction, a coefficient of 9.6 mM^−1^ cm^−1^ was used. The activity of soluble (SPO) and ionic (IPO) peroxidases (EC 1.11.1.7) was measured following the extraction methods and procedures of Ghanati et al. (2002). The activities of soluble peroxidase (SPO) and ionic peroxidase (IPO) were performed following the increase in absorbance at 470 nm using 168 mM guaiacol in 100 mM K-phosphate buffer and 30 mM H_2_O_2_. The change in absorbance was modified to a unit with an extinction coefficient of 26.6 mM^−1^ cm^−1^.

Phenylalanine ammonia-lyase (PAL; EC4.3.1.5) activity was experienced according to Havir and Hanson (2002). The enzyme extract was incubated in a mixture of borate buffer and phenylalanine solution for 1 h at 30 °C; HCl was introduced to stop the reaction. The production of trans-cinnamic acid content was estimated at 290 nm, and the enzyme activity was expressed as μmol mg^−1^ protein^−1^ min. Polyphenol oxidase (PPO; EC1.10.3.1) activity was detected by Lavid et al. (2001) protocol. The purpurogallin production was monitored at 495 nm, and the enzyme activity was expressed in U mg^−1^ protein^−1^ min^−1^.

### Estimation of ascorbate and reduced glutathione

Ascorbic acid (AsA) was determined according to Jagota and Dani (1982). First, leaf samples (0.2 g) were ground with liquid N_2_ and suspended in 2 mL of 5% TCA. Next, the homogenate was centrifuged at 10,000 × g for 15 min at 5 °C. Next, AsA extraction solution was mixed with 10% TCA, which was vigorously shaken and then placed in an ice bath for 5 min. Next, 0.5 ml of the extract was diluted to 2.0 mL using distilled water, and 0.2 mL of diluted Folin-Ciocaiteu reagent was added to the previous mixture, and the absorbance of the blue color developed was measured after 10 min at 760 nm. Finally, the AsA content was calculated using a standard curve of ascorbic acid.

For determination of reduced glutathione (GSH), fresh (100 mg) leaf tissue was macerated in phosphate buffer (pH 8.0) followed by centrifugation for 15 min at 3000 × g. Five hundred milliliters of supernatant and 500 µL of 5,5-dithiobis-2-nitrobenzoic acid were mixed and read at 412 nm after 10 min. The standard curve of GSH was used for the calculation (Ellman 1959).

### Extraction and evaluation of α-tocopherol and lignin content

The α-tocopherol was measured based on (Kivçak and Mert 2001). Fresh leaves were homogenized in prechilled chloroform. To 1 ml of extract, 1 ml of 2,2- dipyridyl, and then 1 ml ferric chloride reagent was mixed and shaken for 10 s. The absorbance of the mixture was read at 522 nm in 50 s after adding the ferric chloride. The *α*-tocopherol content in the extracts was calculated using *α*-tocopherol as a standard curve.

Lignin content was measured according to the method of Doster and Bostock (1988). Leaf powder (0.1 g) was suspended with 0.5 mL ethanol and left for 15 min. After centrifugation, the pellet was dried at room temperature for 12 h. Thioglycolic acid and HCl were mixed with the dried pellet and placed in a water bath at 95 °C for 6 h. The acid-treated pellet was washed with sterile water and re-suspended in NaOH (1 N). The above alkali mixture was stored at room temperature for 12 h with shaking and then centrifuged at room temperature. Concentrated HCl was mixed with supernatant and incubated at 4 °C for 12 h to precipitate the lignin. The lignin was collected by centrifugation, and then the pellet was dissolved in NaOH. The lignin content was measured at 280 nm, and lignin content was expressed as mg g^−1^ of fresh leaf material.

### Statistical analyses

Data were handled and presented in tables and figures as well as checked for normality using Shapiro–Wilk normality testing. Accordingly, data were parametric (*p* > 0.05), the whole data were analyzed statistically by analysis of variance (ANOVA), both one and two-way at 0.05 level. Duncan’s Multiple Range test (DMRTs) was used to further compare between subgroups at *p* ˂ 0.05. Multivariate analysis of variance (MANOVA) was applied to all physiological trait data. Minitab Software performed principal component analysis (PCA).

## Experimental results

### Effect of UVC with different Kn doses on growth characters: examining the best Kn concentration

The data represented in Tables 2 and 3 revealed that several growth parameters, including shoot and root length, fresh weight, and dry weight of shoot and root, have been proposed concerning the impact of UVC and Kn treatments along with one-way ANOVA. UVC induced a highly significant (*p* ˂ 0.001***) decrease in shoot length (42.0%), root length (35.65%), shoot FW (46.25%), root FW (26.53%), shoot DW (33.91%), and root DW by 45.0% as compared to control plants according to DMRTs, where, means with different letters are significantly different. Treatment of UVC-stressed plants with different Kn concentrations (0.05, 0.1, 1.0, 2.0, and 3.0 mM) increased various growth characters. The shoot length (SL) significantly (*p* < 0.05) increased by 13.82 and 17.42% for 0.05 and 0.1 mM Kn application. The highest increase was noticed at the level of 1 mM Kn, where SL increased by 30.81%, whereas, 14.00 and 11.01% increase was detected for 2 and 3 mM Kn spraying level. Root length also showed a maximum increase of 30.88% for 1 mM Kn treatment. The other doses 0.05, 0.1, and 2 mM Kn increased RL by 12.47, 0.1 by 17.65, and 11.14, respectively. The 3 mM Kn treatment remained ineffective in increasing the RL of the stressed plants. The highest increase of 50.65% was noticed by 1 mM Kn treatment in the FW of the shoot. The levels of 0.05 mM Kn increased the shoot FW by 27.11%, 0.1 mM Kn by 33.40%, 2 mM Kn by 37.68%, and 3 mM Kn by 38.44% compared to UVC -exposed plants. Root FW showed a non-significant trend under all Kn doses. Taking into consideration DW of the shoot, the level of 0.05 mM Kn caused a significant increase of 27.61%, 0.1 mM Kn, and 2 mM Kn each by 37.70%; however, the highest growth of 61.80% was noticed by 1 mM Kn treatment. Root DW was maximally (53.19%) increased by 1 mM Kn supply. A significant increase by 31.25% by 0.05 mM Kn, 33.33% by 0.1 mM Kn, 43.58% by 2 mM Kn, and 29.03% by 3 mM of Kn doses was observed compared to alone UVC-treated plants (Tables 2 and 3). Thus, from the above results, it was well established that 1 mM Kn dose was the most optimum for enhancing tomato growth traits. Therefore, only this dose (1 mM) was used for further experiments.

### Effect of Kn applied pre- and post-exposure to UVC in growth characters

**Fig. 1:**
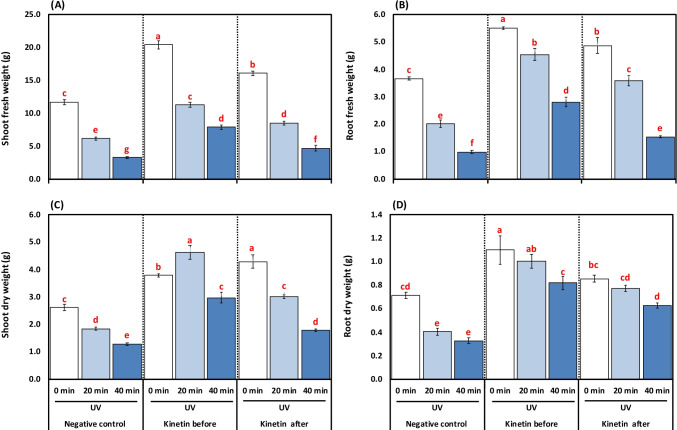
**A-D**. Effects of different kinetin (Kn) doses and UVC on (**A**) shoot fresh weight (g); (**B**) root fresh weight (g); shoot dry weight (g); and (**D**) root dry weight (g) of tomato plants exposed to UVC radiation. Means followed by different letters indicate significant differences among treatment-based Duncan’s Multiple Range Tests (DMRTs) at *p* < 0.05

Increasing the duration of UVC exposure from 20 to 40 min significantly decreased the SFW by 47.26 to 71.40% compared to control plants, respectively. Kn application before UVC exposure increased SFW by 45.39 and 58.15%, respectively, concerning alone 20- and 40-min UVC radiation. A significant (*p* < 0.05) increase in SFW by 27.75 and 29.42%, respectively, was noticed for plants that received Kn after UVC treatment as compared to alone 20- and 40-min UVC radiation (Fig. 1A).

Two-way ANOVA presented in Table 4 revealed that there was a highly significant (*p* < 0.001) effect of Kn or UVC, or interaction between both treatments on growth parameters, e.g., SFW, RFW, SDW, and RDW (Table 4; Fig. 1A-D).

The RFW decreased with increasing (20 to 40 min) duration of UVC radiation by 45.23 and 73.02%, respectively, compared to control plants. On the other hand, Kn application before UVC treatment significantly increased RFW by 55.82 and 64.76%, compared to increments by 44.16 and 35.71%, respectively, for plants that received Kn after UVC exposure compared to alone 20- and 40-min UVC radiation (Fig. 1B).

The SDW was significantly decreased by increasing the duration of UVC radiation (20 to 40 min) by 29.77 and 51.14%, respectively, compared to control plants. The Kn application before and after UVC radiation increased SDW by 60.25 and 57.04%, as well as 38.87 and 28.08%, respectively in SDW compared to alone 20- and 40-min UVC radiation (Fig. 1C).

The 20 to 40 min UVC radiation decreased RDW by 43.66 and 54.92%, respectively, compared to the control group. Kn before UVC treatment supply caused a significant increase in RDW by 60.00 and 60.97%, respectively, concerning 20- and 40-min UVC radiation. An increase of 48.05 and 48.38% was noticed in RDW when Kn was given after UVC radiation compared to plants that received UVC radiation for 20 and 40 min (Fig. 1D).

### Effect of treating plants with Kn under UVC on contents of chlorophyll a, b, carotenoids, and chlorophyll stability index

**Fig. 2:**
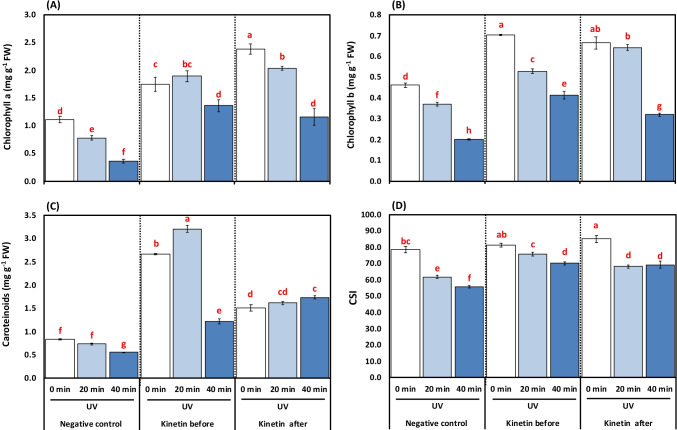
**A-D**. Effects of different kinetin (Kn) doses and UVC on (**A**) chlorophyll *a* (mg g^−1^ FW); (**B**) chlorophyll *b* (mg g^−1^ FW); (**C**) carotenoids (mg g^−1^ FW); and (**D**) chlorophyll stability index (CSI, %) of tomato plants exposed to UVC radiation. Means followed by different letters indicate significant differences among treatment-based Duncan’s Multiple Range Tests (DMRTs) at *p* < 0.05

One of the remarkable outcomes representing the impacts of Kn and their interaction (UVC x Kn) on photosynthetic pigments and chlorophyll stability index (CSI) (Table 4; Fig. 2A-D) as analyzed by 2-way-ANOVA. The 20 to 40 min UVC exposure dose significantly decreased the *Chl a* and *b* contents by 29.72%, 67.56%, and 19.56% and 56.52%, respectively, concerning control plants. On the contrary, Kn application before and after UVC radiation increased Chl a by 58.73% and 73.52% as well as 61.76 and 68.96%, respectively, concerning 20- and 40-min treatments. Likely, the Chl b was stimulated by 30.18 and 51.21, as well as 42.18 and 37.50%, respectively, by Kn application before and after UVC exposure relative to 20- and 40-min UVC (Fig. 2A, B).

Carotenoids and CSI also showed a significant decreasing trend in response to UVC exposure for 20 to 40 min by 12.04% and 33.73% as well as 21.27% and 28.94%, respectively, compared to control plants. The Kn application before UVC radiation increased carotenoids by 77.25 and 54.91%, respectively, concerning alone 20- and 40-min UVC radiation. An increase of 54.93% and 68.20%, respectively, was observed after Kn-treated plants compared to 20- and 40-min UVC treatments. The CSI was increased by 18.37, 79.44, 9.41, and 19.52%, respectively, by Kn application before and after UVC exposure concerning their alone treatments (Fig. 2C, D).

### Effect of Kn application and UVC on oxidative stress biomarkers

**Fig. 3:**
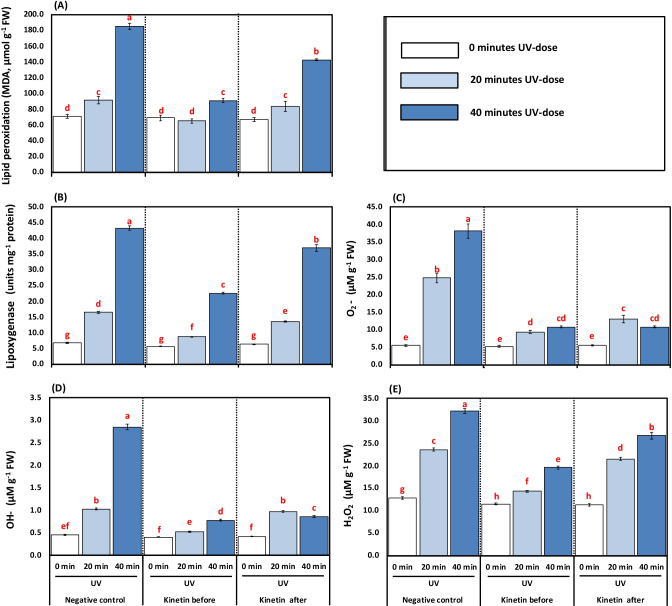
**A-E**. Effects of different kinetin (Kn) doses and UVC on oxidative stress biomarkers represented as (**A**) lipid peroxidation (MDA, μM g^−1^ FW); (**B**) lipoxygenase activity (units mg^−1^ protein); (**C**) superoxide anion (O_2_^**●─**^, μg g^−1^ FW); (**D**) hydroxyl radical (^•^OH, μM g^−1^ FW); and (**E**) hydrogen peroxide concentration (H_2_O_2_, μM g^−1^ FW) of tomato plants exposed to UVC radiation. Means followed by different letters indicate significant differences among treatment-based Duncan’s Multiple Range Tests (DMRTs) at *p* < 0.05

Concerning the results of this experiment, some important observations may be made for the development of MDA, lipoxygenase, and ROS including, H_2_O_2,_ OH^●─^, and O_2_^●─^ in tomato plants treated with Kn in the absence or presence of UVC stress are presented in (Table 4 and Fig. 3). The exposure of tomato plants to 20 and 40 min of UVC radiation significantly increased the MDA and lipoxygenase activity by 22.66, 59.07, and 61.84, 84.44% over their respective control plants (Fig. 3A, B). Treating plants with Kn before UVC caused a dramatic decrease in MDA content and lipoxygenase activity by 28.70, 50.90, and 47.19, 47.91%, respectively, compared to alone 20- and 40-min UVC radiation treatments. While Kn spraying after UVC treatments exhibited a further reduction of MDA content and lipoxygenase activity by 8.38, 23.07, and 17.60, 14.53%, respectively, compared to their corresponding 20- and 40-min UVC treatments.

The contents of O_2_^●─^ and ^●^OH were found to be enhanced in response to 20- and 40-min exposure to UVC by 77.93, 55.88, 45.49 and 85.69, 84.15, 60.08% % over their respective control plants, respectively (Fig. 3C, D). On the other hand, using Kn as a spraying agent before UVC illumination caused a significant decrease in O_2_^●─^ by 62.16 and 71.72% compared to alone 20- and 40-min UVC radiation treatments. Furthermore, the Kn application after UVC treatments imposed a significant reduction in O_2_^●─^ by 47.29 and 71.75. A similar trend was also followed in the case of ^•^OH in which Kn before and after UVC treatments showed a reduction by 49.01, 72.88, and 4.90 and 70.09% respectively compared to their alone 20- and 40-min UVC treatments. The Kn supply before and after UVC radiation also reduced the H_2_O_2_ by 39.12 and 39.14% as well as 8.66 and 17.05%, respectively, compared to solely application of UVC for 20 and 40 min (Fig. 3E).

### Effect of Kn with graded durations of UVC on organic solutes and secondary metabolites

**Fig. 4:**
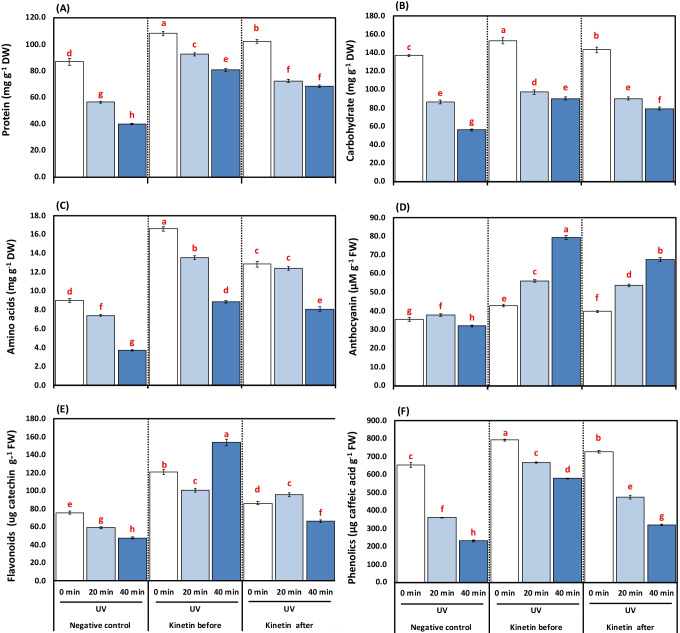
**A-F**. Effects of different kinetin (Kn) doses and UVC on organic solutes represented as (**A**) protein (mg g^−1^ DW); (**B**) carbohydrate content (mg g^−1^ DW); (**C**) amino acids (mg g^−1^ DW); (**D**) anthocyanin (μM g^−1^ FW); (**E**) flavonoids (mg g^−1^ FW); and (**E**) phenolics (mg g^−1^ FW) of tomato plants exposed to UVC radiation. Means followed by different letters indicate significant differences among treatment-based Duncan’s Multiple Range Tests (DMRTs) at *p* < 0.05

Regarding the major organic solutes of tomato plants exposed to 20- and 40-min UVC, protein content attenuated dramatically by increasing the duration of exposure by 35.02 and 53.84%, respectively, relative to the control plants. On the other hand, plants had given Kn before UVC improved the content of proteins by 39.05 and 50.34% respectively as compared to alone 20- and 40-min UVC treatment, as well as 22.19% and 41.60% for UVC-stressed plants, received Kn after illumination, respectively (Fig. 4A).

Exposure of tomato plants to 20 min and 40 UVC significantly decreased the carbohydrate and amino acid by 36.76%, 17.68%, and 59.10%, 58.73% compared with non-illuminated plants. Notwithstanding, plants’ exposed to Kn before UVC caused a significant increase by 10.82, 45.30, and 37.92, 58.17% respectively for 20- and 40-min UVC radiation treatments compared to 4.02%, 40.27%, and 29.29%, 54.02%, respectively, for plants received Kn after UVC application (Fig. 4B and C).

Secondary metabolites downregulated highly significantly by UVC stress with the most drastic effect denoted for high exposure time in terms of anthocyanins (reduced only for UVC exposure time 40 min by 12% and accumulated by 11.40% for 20 min of UVC relative to control), flavonoids (22.08 and 37.17%), and phenolics (44.68 and 64.60%) by UVC stress (Fig. 4D-F). Before UVC treatments, the Kn application imposed a significant upregulation of secondary metabolism where anthocyanins accumulated by 32.48 and 59.67%, flavonoids by 41.43 and 69.07%, and phenolics by (45.76 and 60.17%) compared to the non-sprayed plants received UVC for 20- and 40-min UVC treatments. On the other hand, Kn spraying after UVC treatments showed a significant (*p* < 0.05) increase in anthocyanins and flavonoids by 29.43, 52.73, 38.50, and 28.16%, as well as 23.66 and 27.87%, respectively as compared to their alone 20- and 40-min UVC treatment (Fig. 4D-F).

### Effect of Kn and UVC on antioxidant enzyme activities

**Fig. 5:**
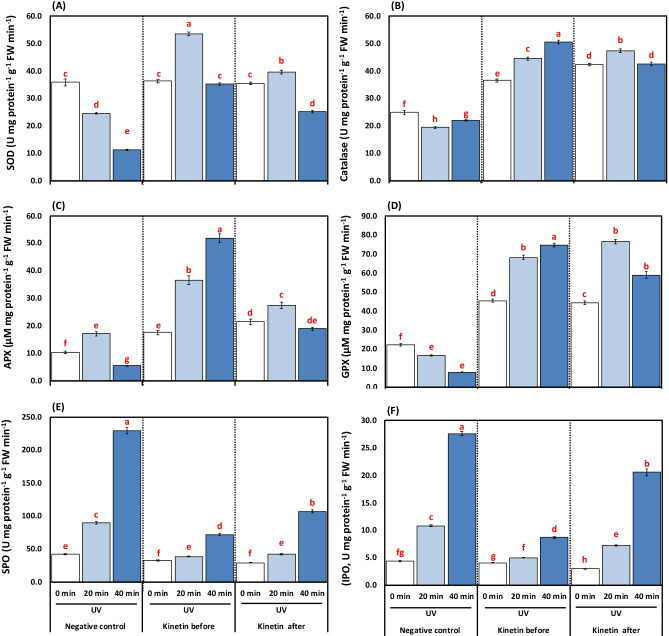
**A-F**. Effects of different kinetin (Kn) doses and UVC on enzymatic antioxidants represented as (**A**) superoxide dismutase (SOD, U mg protein^−1^ g^−1^ FW min^−1^); (**B**) catalase (CAT, U mg protein^−1^ g^−1^ FW min^−1^); (**C**) ascorbate peroxidase (APX, μM mg protein^−1^ g^−1^ FW min^−1^); (**D**) glutathione peroxidase (GPX, μM mg protein^−1^ g^−1^ FW min^−1^); (**E**) soluble peroxidase (SPO, U mg protein^−1^ g^−1^ FW min^−1^); and (**F**) ionic peroxidase (IPO, U mg protein^−1^ g^−1^ FW min^−1^) of tomato plants exposed to UVC radiation. Means followed by different letters indicate significant differences among treatment-based Duncan’s Multiple Range Tests (DMRTs) at *p* < 0.05

The application of 20- and 40-min UVC treatments induced a significant partial deactivation of SOD and CAT activities by 31.29 and 21.99% as well as 68.37 and 11.31%, respectively, over the control plants (Fig. 5A, B). However, external application of the Kn recovered the damaging impacts of UVC, whatever was applied before or after stress. In this regard, SOD activity increased significantly by 53.99 and 67.83, CAT by 25.04 and 56.11% for Kn sprayed plants before UVC treatments and by 37.75 and 55.04 as well as 58.81 and 47.99%, respectively in plants of Kn after UVC treatments concerning their respective exposure durations 20 and 40 min of UVC treatments (Fig. 5A, B).

In APX activity, the doses of 20- and 40-min UVC treatments caused a significant increase and decreased by 38.97 and 46.10%, respectively, compared to control plants (Fig. 5C). On the other hand, GPX activity was significantly reduced by 24.76 and 64.53%, respectively, by the 20- and 40-min UVC treatments doses compared to control plants. APX and GPX activities showed a significant increase of 53.07 and 89.08, as well as 75.40 and 89.40% in Kn, treated plants before UVC stress and by 37.33, 70.22, and 78.07 and 86.60%, respectively in plants received Kn after UVC stress concerning their alone 20- and 40-min UVC treatments (Fig. 5D).

The ionic and soluble POX activities were elevated significantly by 59.02 and 53.07 as well as 83.89 and 81.67% for exposure durations 20 and 40 min of UVC treatments, respectively, compared to control plants. For Kn application before UVC treatment, ionic peroxidase maximized by 53.59, 68.44, and that of soluble peroxidase by 56.47 and 68.62% respectively, and in plants of Kn after UVC treatments, ionic POX increased by 32.78, 25.55, and soluble POX by 52.79 and 53.19%; respectively over their alone 20- and 40-min UVC treatments (Fig. 5E, F).

**Fig. 6:**
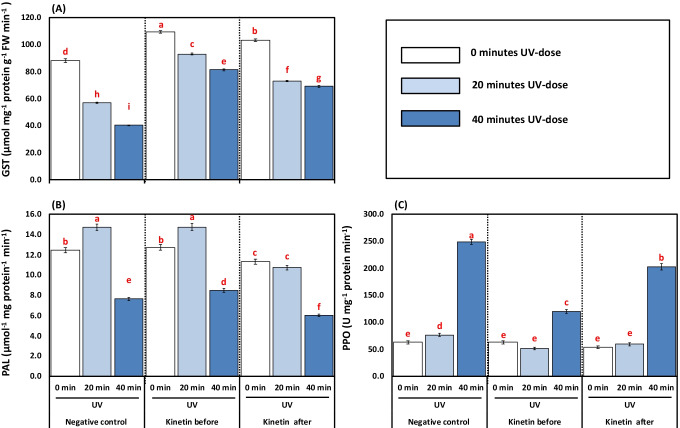
**A–C** Effects of different kinetin (Kn) doses and UVC on enzymatic antioxidants represented as (**A**) glutathione-S-transferase (GST, μmol mg^−1^ protei ng^−1^ FW min^−1^); (**B**) phenylalanine ammonia-lyase (PAL, μmol^−1^ mg protein^−1^ min^−1^); and (**C**) polyphenol oxidase (PPO, U mg^−1^protein min^−1^) of tomato plants exposed to UVC radiation. Means followed by different letters indicate significant differences among treatments based on Duncan’s Multiple Range Tests (DMRTs) at *p* < 0.05

A significant decrease of 31.88 and 48.28% in GST activity was observed by the 20- and 40-min UVC treatments. Nevertheless, GST activity increased significantly by 43.93, 50.92, and 25.37, 40.31% respectively in Kn before and after UVC treatments compared to their alone 20- and 40-min UVC treatments (Fig. 6A).

The 20-min exposure time of UVC imposed a significant stimulation of PAL activity by 15.35, but a higher duration of 40 min decreased its activity by 38.52% concerning the control plants (Fig. 6B). The Kn before UVC treatment increased the PAL activity by 0.20 and 9.88%, respectively, compared to 20- and 40-min UVC treatments. In contrast, the Kn after UVC treatments imposed a significant decrease in PAL activity by 26.97 and 21.27%, respectively, compared to their 20- and 40-min UVC treatment (Fig. 6B).

Plants treated with 20- and 40-min UVC treatments significantly increased the PPO activity by 17.72 and 74.47% compared to control plants. However, a declining trend was noticed when plants were given Kn spraying before UVC treatment by 32.44, 51.41%, and 22.54, 19.31% respectively compared to their alone 20- and 40-min UVC treatment (Fig. 6C).

### Effect of Kn and UVC on non-enzymatic antioxidants, proline, and lignin

**Fig. 7:**
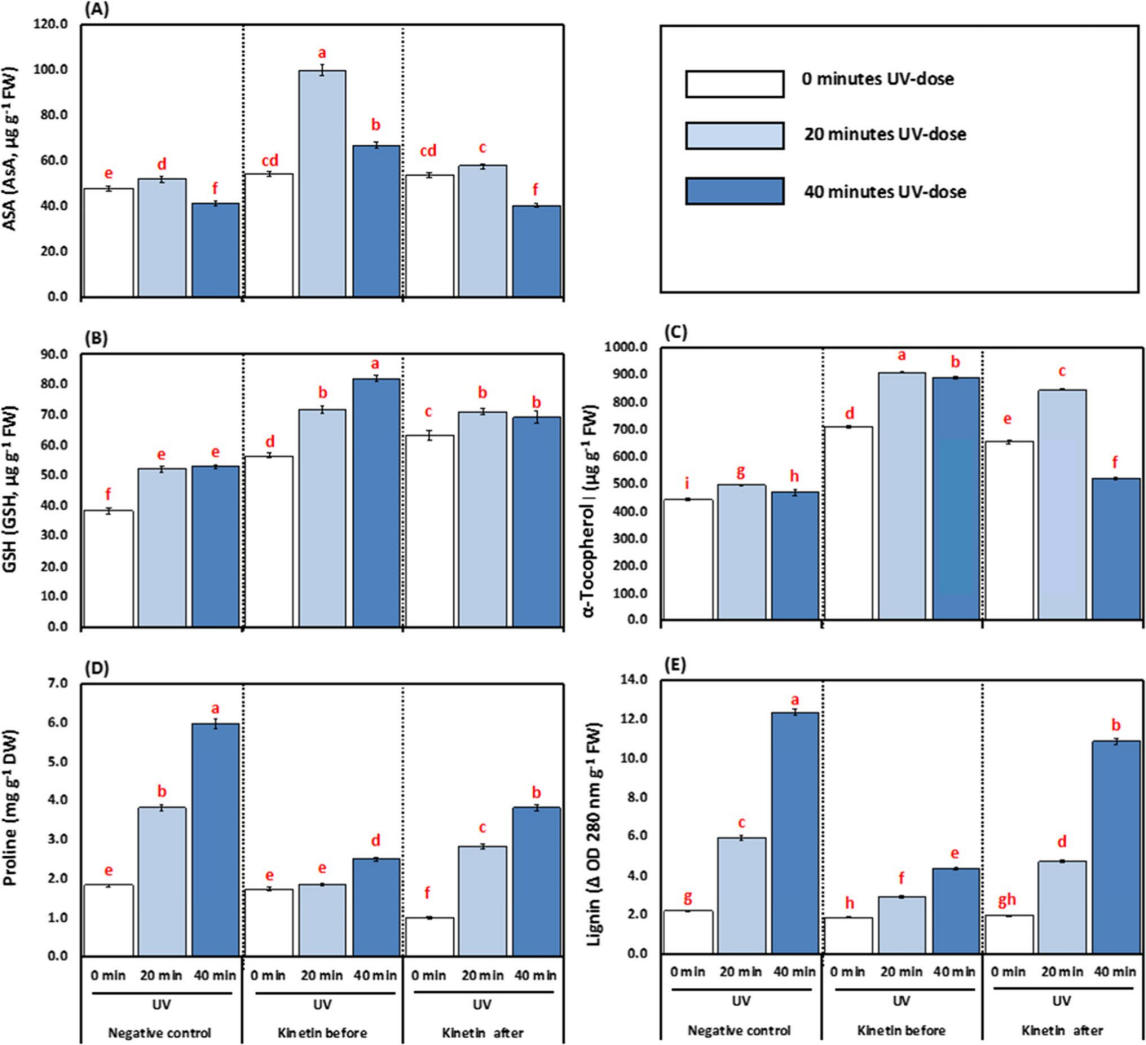
**A-E** Effects of different kinetin (Kn) doses and UVC on non-enzymatic antioxidants represented as (**A**) ascorbate (AsA, μg g^−1^ FW); (**B**) reduced glutathione (GSH, μg g^−1^ FW); (**C**) α-tocopherol (μg g^−1^ FW); (**D**) Proline (mg g^−1^ DW); (**E**) Lignin (Δ OD 280 nm g^−1^ FW) of tomato plants exposed to UVC radiation. Means followed by different letters indicate significant differences among treatment-based Duncan’s Multiple Range Tests (DMRTs) at *p* < 0.05

Plants exposed to 20- and 40-min UVC recorded a significant increase and decrease of AsA content by 7.94 and 13.58%, respectively, over the control plants. While, the plants treated with Kn before UVC increased by 52.00 and 38.16%, respectively, compared to 20- and 40-min treatments. The Kn after UVC treatment 20 min increased the AsA content by 9.94 but decreased by 1.70% at 40 min compared to the 20- and 40-min UVC treatments (Fig. 7A).

The GSH in tomato plants was significantly increased in response to 20- and 40-min UVC treatments by 26.13 and 27.26% over the control. However, exposure to Kn before UVC resulted in a significant increase of 27.13 and 35.38%, respectively, concerning 20- and 40-min UVC treatments. Nonetheless, the Kn after UVC treatments increased the GSH by 26.55 and 23.37%, respectively, over the 20- and 40-min UVC treatment (Fig. 7B).

The α-tocopherol was increased by 20- and 40-min UVC treatments by 10.48 and 5.61% concerning control. The Kn before UVC treatments increased the α-tocopherol by 45.28 and 47.15%%, respectively, compared to the 20- and 40-min UVC treatments. The Kn after UVC treatments also showed significant increments by 41.25 and 9.92%, respectively, concerning 20- and 40-min UVC treatment (Fig. 7C).

Tomato plants exposed to 20- and 40-min UVC showed a significant increment in proline content by 52.21 and 69.39% over the control. On the other hand, plants treated with Kn before UVC treatment showed a decrease of 51.95 and 58.36%, respectively, compared to 20- and 40-min treatments. Also, the application of Kn after UVC decreased proline by 26.10 and 56.54% respectively over the alone 20- and 40-min UVC treatments (Fig. 7D).

The lignin content of tomato plants exposed to 20- and 40-min UVC showed a significant increase of 63.02 and 82.24% over the control. On the other hand, plants treated with Kn before UVC treatments decreased lignin content by 50.92 and 64.72%, respectively, compared to the 20- and 40-min treatments. Also, Kn after UVC treatments decreased lignin by 20.84 and 11.94%, respectively, compared to the alone 20- and 40-min UVC treatments (Fig. 7E).

**Fig. 8:**
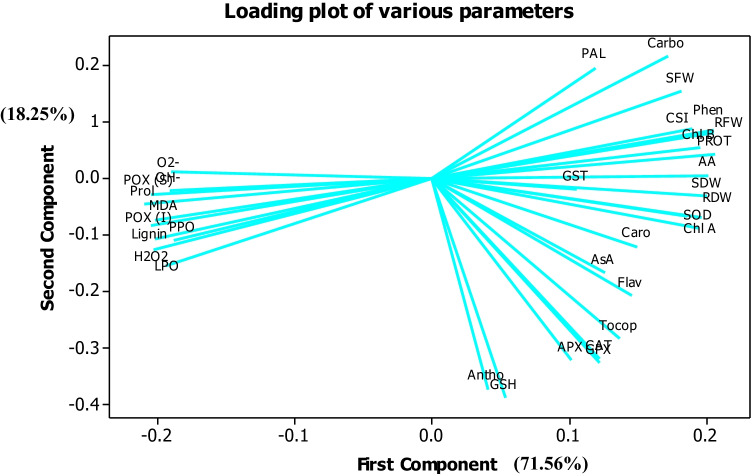
PCA showing the loading plot of various parameters

A PCA was constructed to know the amount of data variability and interaction of various variables. Figure 8 showed the loading plot of multiple variables and indicated that growth, photosynthetic pigments, antioxidants enzymes (POX), and secondary metabolites were positively correlated with each other and negatively with ROS, PPO, LPO, MDA, proline, and POX activity.

## Discussion

The present study included two parts; the first part included applying different doses of Kn to select the best concentration of Kn in easing the UVC induced stress on tomato plants. In the second subsequent study, the efficacy of the pre-and post-application of Kn on UVC stressed plants was attempted to unravel the underlying mechanisms for UVC stress tolerance in tomato plants. In the present study, the UVC restricts the growth characters reflected in the decreased length and fresh and dry weight of the studied plant's organs (Tables 2 and 3, and Fig. 1). Similar results are also confirmed by Yang et al. (2004), and Vaseva et al. (2006). The possible reason for reduced growth attributes may be due to UV rays’ impact on the endogenous cytokinin levels, significantly decreasing plants’ growth. Similar UV rays mediated decrease in growth traits of *Solanum lycopersicum* (Bashri et al. 2018; Singh et al. 2019), maize (An et al. 2005), and lettuce (Krizek et al. 1998) plants has also been reported. In plants, UV rays decrease the growth characters by reducing the expression of growth-regulating factors and gibberellin levels (Fina et al. 2017). In addition, the decreased growth attributes under UV stress could be due to the inhibition in photosynthetic activity, decreased light-harvesting pigments: Chl *a*, Chl *b,* and carotenoids contents (Ma et al. 2018), and also by enhanced oxidative stress by the accumulation of various types of ROS in tissues (El-Sheshtawy et al. 2021; Maksoud et al. 2022; Sofy et al. 2021b; Waseem et al. 2021).

Before or after stress, the treatment of Kn whatever ameliorated the UVC-induced disturbances in the tomato seedlings’ growth. Such increment could be ascribed to improved photosynthetic pigment activity and possibly increased UV-absorbing pigments. Similar results under Kn application and UV rays stress in *Astragaluss missouriensis* (Ionkova 2009) and *Solanum lycopersicum* (Singh et al. 2019) have been reported; they agree with our present results. Thus, it may be proposed that the exogenous Kn application supported the tomato seedlings’ growth even under short (20 min) or extended (40 min) UVC stress.

In the present study, the photosynthetic pigments (*Chl a*, *b,* and Car) and CSI of tomato seedlings were found to be decreased under UVC exposures (Fig. 2a, b, c, and d). The decrement in pigment contents under UV rays significantly induces photosynthetic capacity inhibition of chloroplasts (Abdel-Kader et al. 2007; Amal et al. 2006; Kakani et al. 2004; Khan et al. 2017). Singh et al. (2019) reported that UV radiation-mediated decrements in *Chl* pigment contents, photosynthesis-evolving activity, enzyme activity, and PS II photochemistry were the major causes of retardation in the growth characters, as has been reported by the present work. It has been reported that UV radiation-mediated reduction in photosynthetic pigments was attributed to the inhibition of chlorophyll biosynthesis pathway-related enzymes and their precursors such as protochlorophyllide and disorientation of thylakoid membranes (Akladious and Mohamed 2017; Alharbi et al. 2021; Khaleil et al. 2020; Ranjbarfordoei et al. 2011; Sharaf et al. 2009). The results conform to the findings of Wang et al. (2010), stating that UV radiation mediated a decrease in Chl and Car contents of *Wolffia arrhiza*. Interestingly, Kn spraying before or after UVC stress upregulated the biosynthesis of photosynthetic pigments and their stability index. This could be due to the cytokinin-mediated enhancement in the tetrapyrrole ring’s biosynthesis, which restores and enhances the photosynthesis complexes, thereby increasing the activity of photosynthesis (Ghonaim et al. 2021; Jahan et al. 2021; Mohamed and Abd-El Hameed 2014). A similar Kn mediated increase in photosynthetic pigments under UV stress has earlier been reported by Bashri et al. (2018).

The increase in primary and secondary metabolite contents is a characteristic phenomenon under UV stress conditions (Thomas and Puthur 2020). Unlikely, the sensitivity of tomato plants to UVC application is denoted from a substantial decrease in major primary metabolic products as proteins, carbohydrates, and amino acids in the present study (Fig. 4A-F) which could be associated with growth retardation under UVC stress. Thus, UVC exerted its negative effect on plants via induction of early senescing of tomato plants. The application of Kn modulated all the primary metabolites under UVC stress conditions in line with upregulation of the photosynthetic moiety, hence recovering the stressed plants from senescing. In conformity, PCA analysis showed a positive correlation between growth parameters (lengths and weights), pigments, as well as metabolites (proteins, carbohydrates, and amino acids).

The oxidative stress biomarkers were studied to fully understand the effects of UVC radiation doses and possible amelioration by Kn on tomatoes. The MDA content, LOX, and various ROS were accumulated under UV doses in tomato plants (Fig. 3A–D). The increment in oxidative stress biomarkers under UV doses reflects a state of disturbed redox balance. UVC radiation exposure was found to cause a variety of ROS generation in the current study (Fig. 3). In general, enhanced oxidative stress results from normal cellular homeostasis disruption by excess ROS biosynthesis (Abdel Razik et al. 2020; Moustafa-Farag et al. 2020; Shaukat et al. 2013; Soliman et al. 2020b). The excess ROS in plants is known to affect vital physiological processes negatively. In this regard, the overproduction of ROS in plants causes oxidation to proteins, lipids, DNA, and RNA (Bashandy et al. 2020; Sofy et al. 2021c; Soliman et al. 2020a; Zaheer et al. 2020; Zaid and Wani 2019). The enhanced biosynthesis of ROS and lipids’ peroxidation under UV doses causes a state of oxidative stress in tomato plants. The stress state disturbs normal metabolic processes and consequently decreases the growth and photosynthetic pigments in the present study. A similar increase in ROS contents and lipid peroxidation under UV radiation stress has been reported in *Zea mays* seedlings (Singh et al. 2016) which are in agreement with our present results. The excess production of ROS and MDA (Fig. 2A–D) in UVC stressed tomato plants could be due to a reduction in the activities of antioxidant defense enzymes like SOD, CAT, APX, GPX, and GST (Fig. 5A–D). On the other hand, Kn-treated tomato plants showed a reduction in various ROS produced, thereby minimizing the damage of UV stress. The possible effect of Kn is due to its action on the plant antioxidant defense system, which alleviates the damaging impact of various ROS in the present study. This upregulation of the activities of antioxidant enzymes under UVC stress in tomato minimized the damaging impacts on the membrane where lower values of MDA content, lipoxygenase activity, and various forms of ROS. A similar Kn-mediated increase in antioxidant enzymes and a decrease in stress biomarkers have been reported in some plants (Abdelaal et al. 2020; Abu-Elsaoud et al. 2017; Abu-Shahba et al. 2022; El-Beltagi et al. 2019; Singh et al. 2019).

In line with enzymatic antioxidants, the non-enzymatic antioxidants like AsA, GSH, tocopherol, and proline are increased under UV treatments (Fig. 7A–D). The Kn application had an additive effect on these traits except proline. The antioxidants played an essential role in lowering the toxic effect of excess ROS produced inside the cells. The checked balance of ROS production under UV stress in plants given Kn helped tomato plants maintain redox homeostasis, thus enabling plant processes’ normal functioning. In line with our experiment, Gao and Zhang (2008) have studied the role of AsA under UV stress-induced oxidative stress in AsA deficient (*vtc1*) mutants of *Arabidopsis thaliana*. The significant improvement in non-enzymatic antioxidants along with proline content in Kn-treated plants suggested that these non-enzymatic antioxidants and proline content upregulated by Kn supported plants to regulate the oxidative stress under UVC toxicity as indicated by better growth performance and reduced ROS content in Kn-treated than non-Kn ones (Table 2 and Figs. 1 and 3). A similar increase in maize redox environment under UV stress under the phytohormone application has been reported (Singh et al. 2019). Thus, our results demonstrated that both ascorbate and tocopherol could play crucial roles in plant defense against UVC radiation. Yao et al. (2015) showed that the ascorbate could profoundly affect plant sensitivity to UV-B radiation through three biological processes: (1) synthesizing phenylpropanoid and flavonoid compounds; (2) scavenging the ROS produced in photosynthesis; (3) dissipating excess photons by NPQ. PAL is a key enzyme in the phenylpropanoid pathway, producing flavonoids, anthocyanins, and many other related metabolites. In the present study, the PAL activity is decreased by UV stress (Fig. 6B), but PPO is increased (Fig. 6C). Consequently, the decrease in these phenolic products may be attributed to the decline in their biosynthesizing-enzymes (PAL) activity and enhancing phenol-oxidizing enzyme (PPO).

In the present work, anthocyanins, flavonoids, and phenolic compounds were negatively affected by UVC stress. These secondary products generally act as screen savers against bad radiation, thus rendering tomato plants sensitive even to a short duration of UVC stress (20 min). Interestingly, Kn application increased the secondary metabolites on the one hand and triggered an increase in enzymes’ activity (PAL) on the other hand. A similar effect of Kn on increasing anthocyanins, flavonoids, phenols, and PAL activity has been observed previously in tomatoes under UV stress (Singh et al. 2019). It is worth mentioning that a central relationship between PAL activity and flavonoid has been observed in *Spirodela intermedia* using PAL inhibitors (Abd El-Rahman and Mohamed 2014; Gitz 2004). It was found that the PAL-inhibitor reduced the synthesis of flavonoids, thus making *Spirodela intermedia* more responsive to UV stress. Phenylopronoid and flavonoid biomolecules have shown a nice pattern and can protect cellular components from ultraviolet radiation, as confirmed by Frohnmeyer and Staiger (2003). In the mesophyll cell, the leaves’ epidermis accumulates flavonoids which are UV-absorbing pigments and are considered as the first line of defense when exposed to UV-B stress by scavenging the singlet oxygen, thereby preserving the chloroplast envelope integrity (Agati et al. 2011). Also, the enrichment of anthocyanins could partly cancel out the adverse effects of UV-B irradiation (Singh et al. 2019). A secondary metabolite associated with PAL products, lignin content, was increased by UV treatments (Fig. 7E). The high lignin content might be one of the protective mechanisms adopted by tomatoes under UVC radiation stress to avoid damage. Thus, the plants tend to reduce their content under the Kn application, where other defencing mechanisms were instigated. Gathering together, the mechanistic role of Kn under UVC stress could be associated to the accumulation of PAL activity, which accumulates anthocyanin, flavonoids, and phenolics by the upregulatory role of ascorbate and tocopherol.

A final validation in testing Kn treatments’ effectiveness in alleviating UVC stress damages in the present study was documented from PCA analysis. According to the PCA loading plot (Fig. 8), growth, photosynthetic pigments, and primary and secondary metabolites were in opposite directions with ROS and MDA contents. This showed that the plant growth under UVC stress was attenuated with ROS production, which was the major stress marker of UVC radiation. The antioxidants (enzymatic and non-enzymatic) and secondary metabolites, osmolytes, were the major defending mechanism that exerts a regulation under Kn-UV interaction. The loading plot of the PCA showed that antioxidants and osmolyte characters lay between plant growth and oxidative stress biomarkers.

## Conclusion

The present study revealed that UV radiations caused oxidative stress that decreased growth and induced alterations in physio-biochemical attributes. In contrast, the UVC mediated decrement in growth traits, photosynthetic pigments, protein content, and primary metabolites was overcome by Kn application. The application of Kn at selected doses minimized the UV-induced oxidative stress and protected tomato plants under UVC. The application of pre-and post-Kn treatment also decreased UVC-induced stress by boosting the primary and secondary metabolites’ status and elevating the plant antioxidant gadgets. Thus, it was concluded that Kn could increase growth and yield through different modes of applications and alleviate UVC-induced ill effects in tomato plants.

## Future outlooks

Although the present study provides new information on the mechanistic role of kinetin under UVC stress, many points should be taken into consideration for further studies:

- Ultrastructural changes of different organs under UVC stress should be documented.
- The correlation of endogenous hormones under UVC should be studied extensively.
- Genes controlling ROS production under UVC stress.
- Gene expression of PAL and other secondary metabolites under UVC-kinetin conditioning.
- Further studies on the interactive effect of UVC and kinetin or other PGR should be done on other crop plants.
- Induction of senescing by UVC and its recovery by kinetin.

**Table 1:**
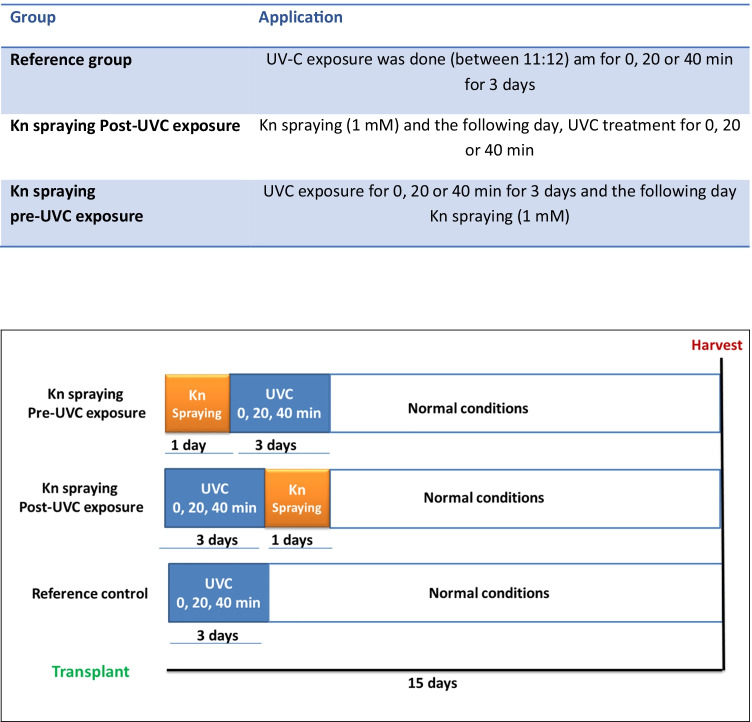
The design of the experiment

**Table 2:** Effects of different kinetin (Kn) doses and UV on (A) shoot length (cm); (B) root length (cm); and shoot fresh weight (g) of tomato plants exposed to UVC radiation. Means followed by different letters indicate significant differences among treatments, based on Duncan’s Multiple Range Tests (DMRTs) at *p* < 0.05

Treatments	Shoot length (cm)	Root length (cm)	Shoot fresh weight (g-FW)
**Control**	29.66 ± 1.19^**a**^	14.50 ± 0.70^**a**^	5.60 ± 056^**a**^
**UV**	17.20 ± 1.04^**d**^	9.33 ± 0.75^**d**^	3.01 ± 0.23^**d**^
**0.05 mM**	19.96 ± 1.02^**c**^	10.66 ± 0.75^**c**^	4.13 ± 0.43^**c**^
**0.1 mM**	20.83 ± 1.26^**c**^	11.33 ± 0.53^**c**^	4.52 ± 0.46^**bc**^
**1 mM**	24.86 ± 1.12^**b**^	13.50 ± 0.93^**b**^	6.10 ± 0.58^**a**^
**2 mM**	20.00 ± 1.00^**c**^	10.50 ± 0.70^**c**^	4.83 ± 0.76^**b**^
**3 mM**	19.33 ± 0.75^**c**^	9.00 ± 0.65^**d**^	4.89 ± 0.60^**b**^
**ANOVA**			
F-ratio	37.48	44.22	27.32
*p*-value	< 0.001***	< 0.001***	< 0.001***

^***^, highly significant at *p* < 0.001

**Table 3:** Effects of different kinetin (Kn) doses and UV on (A) root fresh weight (g/plant); (B) shoot dry weight (g/plant), and root dry weight (g/plant) of tomato plants exposed to UVC radiation. Means followed by different letters indicate significant differences among treatment based Duncan’s Multiple Range Tests (DMRTs) at *p* < 0.05

Treatments	Root fresh weight (g/plant)	Shoot dry weight (g/plant)	Root dry weight (g/plant)
Control	1.47 ± 0.25^**a**^	1.15 ± 0.17^**c**^	0.40 ± 0.40^**b**^
UV	1.08 ± 0.22^**b**^	0.76 ± 0.14^**f**^	0.22 ± 0.22^**d**^
0.05 mM	0.64 ± 0.16^**e**^	1.05 ± 0.16^**d**^	0.32 ± 0.32^**c**^
0.1 mM	0.74 ± 0.17^**d**^	1.22 ± 0.17^**b**^	0.33 ± 0.33^**c**^
1 mM	1.01 ± 0.20^**b**^	1.99 ± 0.20^**a**^	0.47 ± 0.47^**a**^
2 mM	0.83 ± 0.19^**c**^	1.22 ± 0.17^**b**^	0.39 ± 0.39^**b**^
3 mM	0.72 ± 0.22d^**e**^	0.96 ± 0.15^**e**^	0.31 ± 0.31^**c**^
ANOVA			
F-ratio	122.21	492.22	146.65
*p*-value	< 0.001***	< 0.001***	< 0.001***

^***^, highly significant at *p* < 0.001

**Table 4:** Multivariate analysis of variance (MANOVA) of various study variables under the effect of both kinetin and UV and their interaction

Effect	Corrected model		Kinetin		UV		Kinetin * UV	
	**F**	***P***	**F**	***p***	**F**	***P***	**F**	***p***
**Pillai's trace**	**–**	**–**	393.7	< 0.001***	5356.5	< 0.001***	240.1	< 0.001***
**SFW**	218.1	< 0.001***	203.1	< 0.001***	649.9	< 0.001***	9.6	< 0.001***
**RFW**	95.1	< 0.001***	125.3	< 0.001***	248.0	< 0.001***	3.5	0.028*
**SDW**	63.3	< 0.001***	126.1	< 0.001***	91.0	< 0.001***	18.0	< 0.001***
**RDW**	22.3	< 0.001***	63.2	< 0.001***	23.1	< 0.001***	1.5	**0.241 ns**
**Chl-a**	48.7	< 0.001***	123.1	< 0.001***	59.4	< 0.001***	6.2	0.002**
**Chl-b**	141.7	< 0.001***	197.3	< 0.001***	338.3	< 0.001***	15.5	< 0.001***
**Carotenoids**	432.6	< 0.001***	1132	< 0.001***	205.6	< 0.001***	196.1	< 0.001***
**CSI**	42.6	< 0.001***	45.4	< 0.001***	110.0	< 0.001***	7.4	0.001***
**Protein**	229.5	< 0.001***	393.0	< 0.001***	492.4	< 0.001***	16.4	< 0.001***
**Carbohydrates**	236.8	< 0.001***	67.5	< 0.001***	860.0	< 0.001***	9.8	< 0.001***
**Amino**	368.4	< 0.001***	759.9	< 0.001***	677.6	< 0.001***	18.0	< 0.001***
**Proline**	500.1	< 0.001***	593.9	< 0.001***	1071.9	< 0.001***	167.3	< 0.001***
**AsA**	192.5	< 0.001***	372.0	< 0.001***	220.4	< 0.001***	88.9	< 0.001***
**GSH**	117.2	< 0.001***	303.8	< 0.001***	130.3	< 0.001***	17.3	< 0.001***
**Tocopherol**	1281.7	< 0.001***	3696	< 0.001***	677.4	< 0.001***	376.2	< 0.001***
**Anthocyanin**	426.8	< 0.001***	801.6	< 0.001***	514.4	< 0.001***	195.6	< 0.001***
**Flavonoids**	262.8	< 0.001***	774.4	< 0.001***	15.0	< 0.001***	130.8	< 0.001***
**Phenolics**	835.9	< 0.001***	1170	< 0.001***	2011.1	< 0.001***	81.1	< 0.001***
**Lipid peroxidation**	128.3	< 0.001***	95.2	< 0.001***	329.3	< 0.001***	44.4	< 0.001***
**Lipoxygenase**	877.0	< 0.001***	344.1	< 0.001***	2923.2	< 0.001***	120.3	< 0.001***
**Lignin**	1666.2	< 0.001***	1272	< 0.001***	4408.5	< 0.001***	491.6	< 0.001***
**O2-**	146.8	< 0.001***	229.3	< 0.001***	203.3	< 0.001***	77.3	< 0.001***
**OH**	925.8	< 0.001***	1017	< 0.001***	1390.2	< 0.001***	647.7	< 0.001***
**H**_**2**_**O**_**2**_	335.2	< 0.001***	280.1	< 0.001***	946.2	< 0.001***	57.3	< 0.001***
**Catalase**	421.2	< 0.001***	1484	< 0.001***	34.5	< 0.001***	82.9	< 0.001***
**APX**	193.3	< 0.001***	433.1	< 0.001***	95.7	< 0.001***	122.2	< 0.001***
**Ionic peroxidase**	988.0	< 0.001***	726.2	< 0.001***	2560.5	< 0.001***	332.7	< 0.001***
**soluble peroxidase**	944.2	< 0.001***	1055	< 0.001***	1990.7	< 0.001***	365.1	< 0.001***
**Polyphenol oxidase**	408.6	< 0.001***	151.6	< 0.001***	1294.1	< 0.001***	94.4	< 0.001***
**Glutathione perox**	673.3	< 0.001***	2136	< 0.001***	209.6	< 0.001***	173.7	< 0.001***
**Glutathione-s-tr**	0.6	**0.741 ns**	1.5	**0.255 ns**	0.9	**0.423 ns**	0.1	**0.989 ns**
**SOD**	376.8	< 0.001***	652.0	< 0.001***	534.4	< 0.001***	160.3	< 0.001***
**PAL**	156.8	< 0.001***	100.8	< 0.001***	503.5	< 0.001***	11.5	< 0.001***

*NS*, non-significant at *p* > 0.05

^*^, **, ***, significant at *p* < 0.05, < 0.01, < 0.001; respectively

## Data Availability

Not applicable.
